# Perceived stress, sources of stress and coping strategies among undergraduate medical students of Nepal: a cross-sectional study

**DOI:** 10.12688/f1000research.75879.2

**Published:** 2023-09-13

**Authors:** Ujjawal Paudel, Anima Parajuli, Rashmi Shrestha, Shivanee Kumari, Saroj Adhikari Yadav, Kedar Marahatta

**Affiliations:** 1 School of Medicine, Patan Academy of Health Sciences, Kathmandu, Nepal

**Keywords:** stress, coping stress, medical students, medical education, mental health.

## Abstract

**Background:** Medical students are subjected to various stressors throughout their training, which has a considerable impact on their physical and mental health. Some students have positive ways of coping, while others take to maladaptive coping measures. This study aims to assess severity, sources of stress, and coping strategies among medical students of a non-Western low-income country from South Asia.

**Methods:** A self-administered questionnaire-based cross-sectional study was carried out. Demographic variables were collected and stress level was assessed using PSS 14 (Perceived Stress Scale 14). The sources of stress were assessed using MSSQ (Medical Student Stressor Questionnaire) and coping strategies were evaluated using the Brief-Coping Orientation to Problems Experienced 28.

**Results:** The response rate was 95%. The mean PSS score was 27.85. Overall, 55% of students were stressed (male 52%, female 60%), the difference among gender was not significant. Basic science students perceived higher levels of stress than clinical clerkship students. Academic related stressors caused higher stress, whereas other variables caused moderate stress. The major stressors were examinations, inadequate time to study, poor marks, extensive amount of learning content, and the need to performing well in the exam. The five most common coping strategies used were active coping, acceptance, planning, self-distraction, and instrumental support. The least common coping mechanism was substance use. All MSSQ domains positively correlated with the total PSS score. Students with higher PSS scores were likely to use behavioral disengagement, venting, and self-blame as the primary coping strategies.

**Conclusions:** Stress level among the medical students is high and mainly in relation to academia. Inadequate guidance from teachers contributed significantly. Stressed students were likely to use maladaptive coping strategies. Strategies to enhance teacher-student communication and adaptive coping measures should be implemented. Further studies should be done to evaluate the effects of stress on the academic outcomes of students.

## Introduction

Stress is a state of mental or emotional strain or tension resulting from adverse or demanding circumstances. ^1^ Medical students are subjected to various stressors throughout their training, which can have profound effects on their well-being. A systematic review by Liselotte *et al*. showed overall psychological distress consistently higher in medical students than in the general population. ^2^

Studies that have examined sources of stress among medical students generally point to three key areas: academic pressures, social issues, and financial problems. ^3^ These stress and the various ways in which students cope with them are of vital importance as they can have a considerable impact on their physical and mental health, necessitating an exploration of their origin and the coping mechanism employed in response. ^4^ While some students adopt positive ways of coping with stressors like positive reframing, planning, and recreational activities like sports and music, ^5^ while others may opt for maladaptive coping measures like substance abuse. ^6^ Similarly, a study among medical students in North India, which is quite similar to Nepal, showed significant stress among medical students. Worrying about the future was rated the highest by the final year students, faculty shortcomings and insufficient feedback were rated highest by the second-year students, and financial concerns the highest by the first-year students. ^7^ Previous studies investigating the link between stress and academic performance in medical students have shown a negative correlation between the high level of stress and academic performance. ^8^

In the context of medical education, research on the psychological distress experienced by medical students in Nepal remains sparse. While a few studies have been done into this terrain, there scope has been limited. A study was done in Manipal College of Medical Science, Pokhara, Nepal, using the General Health Questionnaire (GHQ). It showed a prevalence of psychological morbidity among medical students of 20.9%. ^9^ The most common causes of stress identified by the students were quality of food in mess, dissatisfaction with lecture classes, and vastness of academic curriculum, and frequency of examinations. ^9^ Another study done in Universal College of Medical Sciences, Bhairahawa, Nepal showed that stress during exams and preparation phase stood out as the stressful period among medical students. ^10^ A high prevalence of depression, anxiety, and burnout was found among medical students and residents in another study in Nepal, the academic-related factors being the main stressor identified in the study. ^11^ These studies, though valuable, fall short of providing a comprehensive exploration of the magnitude of stressors and coping mechanism adopted by medical schools in Nepal.

To address this gap, our study ventures into the domain of Patan Academy of Health Sciences (PAHS), a distinguished medical school adopting innovative teaching-learning approaches. Furthermore, there has been no study on psychological stress among medical students of PAHS. Therefore, we wanted to identify the severity of stress, recognize the sources of stress and explore the coping strategies used, among undergraduate medical students of PAHS, Kathmandu, Nepal. By delving into the unique context of PAHS and its innovative teaching methods, our research not only contributes to the understanding of stress in medical education but also offers valuable insights into the broader spectrum of medical education in Nepal.

## Methods

### Study design, participants, and sample size

This is a cross-sectional study conducted from 15 November 2019 to 14 December 2019 among undergraduate medical students of PAHS, a government medical school of a non-Western low-income country, Nepal. PAHS has adopted several new and innovative approaches in its medical curriculum. These include Problem-Based Learning (PBL) in the first two years of integrated basic science study, and the Clinical Presentation Curriculum (CPC) in third to final years of clinical clerkship.

The study is based on self-administered questionnaires. Written informed consent was obtained from the participants. The study included all medical students of the four batches currently in School of Medicine of PAHS. These batches consist of two groups in initial basic sciences years of medical school and two groups in later clinical clerkship years of medical school. Consenting students were asked to fill out the questionnaire together in class. Students not giving consent and students among the researcher team were excluded from the study. A total of 231 students from four batches were initially considered for inclusion in this study.

As there were no previous estimates of the prevalence of stress among medical students in Nepal, a conservative assumption of a prevalence of 50% was made, with a 10% margin of error, and a 5% level of significance. After adding a 10% non-response rate, the final sample size targeted for the study came out to be 106. To select study participants, a two-stage stratified random sampling method was employed. Stratification was based on the year of study and gender. Within each stratum, students were randomly selected using the lottery method. Out of the 106 sample of randomly selected students, 101 completed the questionnaire and provided consent.

The questionnaires administered to the students consisted of four parts: Demographics, Medical Student Stressor Questionnaire (MSSQ), ^12^ Perceived Stress Scale (PSS), ^13^ and Brief-COPE (Coping Orientation to Problems Experienced). ^14^ All questionnaires were in English language. Ethical approval was taken from the institutional research committee of the Patan Academy of Health Sciences [Ref no: std1506111071] and research was carried out per relevant guidelines and regulations. ^15^

### Description of tools

*Demographic questionnaire*

A demographics questionnaire was developed for this study. It comprised of questions pertaining to various demographic details such as age, gender, address, year of study, weather the participant resided in PAHS hostel or not, etc. These questionnaires were compiled in Microsoft Word (Microsoft Office Professional Plus 2019 version 2112) and discussed in the research group and reviewed by the research advisors to establish content validity. Finalized questionnaire were distributed among all respondents.

*Perceived Stress Scale questionnaire*

Perceived stress level was measured using the PSS-14. ^13^ PSS-14 is a pretested and pre-validated questionnaire designed to assess an individual’s perception of stress. This questionnaire comprises 14 questions with responses varying from “never,” “almost never,” “sometimes,” “fairly often,” and “very often” labeled as 0-4 respectively. It is designed to assess the degree to which participants evaluate the stress levels in their lives over the previous month. ^13^

PSS-14 scores were obtained by reversing the scores on positive items, following a pattern where 0 was converted to 4, 1 to 3, 2 to 2, and so on, and then summing across all 14 items. The scale yields a single score where high scores meant higher perceived levels of stress and lower scores meant lower perceived levels of stress. The PSS-14 has a range of scores from 0 to 56. A cut-off value of 28 (equivalent to 50%) was used, and the scores were labeled as “stressed” or “not stressed” depending on weather their scores exceeded of fell below this threshold, respectively. This specific cut-off value was selected from a similar study done in Pakistan. ^5^

*Medical Student Stress questionnaire*

In this study, the MSSQ was used to assess sources of stress and their severity. ^12^^,^^16^ The MSSQ as well is a pretested and pre-validated questionnaire. It comprises 40 questions. Respondents were asked to rate this set of forty items or stressors using a scale of 0 to 4 where 0 = causing no stress at all, 1 = causing mild stress, 2 = causing moderate stress, 3 = causing high stress, and 4 = causing severe stress. These stressors encompass a wide array of challenges commonly faced by medical students, including examinations, heavy workload, lack of time to review what has been learned, etc.

The MSSQ grouped stressors in six domains based on an underlying theme. ^12^ The six domains are: 1. Academic Related stressors (ARS), 2. Intrapersonal and interpersonal Related Stressors (IRS), 3. Teaching and Learning-related stressors (TLRS), 4. Social Related Stressors (SRS). 5. Drive and desire Related Stressors (DRS), and 6. Group Activities Related Stressors (GARS). ^12^ Total scores in individual domains were calculated by using weighted means. Stressors with scores of 0 – 1.00, 1.01 – 2.00, 2.01 – 3.00, and 3.01 – 4.00 were graded as mild, moderate, high, and severe respectively.

*Brief-COPE questionnaire*

The Brief-COPE-28 scale, a pretested pre-validated questionnaire, was used to assess coping strategies used by students in response to their stress. ^14^ It contains 28 items and is rated on a four-point Likert scale; 1 = I haven’t been doing this at all, 2 = I’ve been doing this a little bit, 3 = I’ve been doing this a medium amount, 4 = I’ve been doing this a lot. In total, this scale covers 14 dimensions. These are grouped into maladaptive coping strategies denial, self-distraction, behavioral disengagement, venting, self-blame, and substance use and adaptive coping strategies active coping, instrumental support, planning, acceptance, emotional support, humor, positive reframing, and religion. ^14^

### Data collection and analysis

A total of 106 students were randomly selected from the sample using two-stage stratified random sampling based on the year of study and gender, followed by the lottery method for each stratum to ensure a representative sample. The selected students were given questionnaires with written instructions. Informed consent was taken from all the participants in written form. The questionnaire was distributed amongst students and the researchers collected the completed questionnaires researchers for analysis.

Data were entered into Microsoft Excel (Microsoft Corporation, 2018, Microsoft Excel) (RRID: SCR_016137). It was analyzed using Statistical Package for Social Sciences (SPSS) 13.0 (SPSS Inc. Released 2005. SPSS for Windows, Version 13.0. Chicago) (RRID: SCR_002865).

To assess the perceived stress levels, the mean score of perceived stress was calculated. The number and percentage of stressed cases were calculated according to demographic variables. Six different domains of MSSQ, i.e. ARS, IRS, TLRS, SRS, DRS and GARS, were calculated. Descriptive statistics were used to measure the severity of stressors. Spearman’s correlation was applied to test the correlation between perceived stress (PSS score) and coping strategies among students. The p-value<0.05 was considered significant.

## Results

Out of initial pool of randomly selected 106 students, one didn’t give consent for the study and four didn’t complete the questionnaire. 101(95%) of original sample filled and submitted the questionnaire which was used for further analysis. The demographic characteristics of students are shown in Table 1. Since four of the students did not fill in the address on the questionnaire, they were omitted from the analysis of this specific question and the remaining 97 were used to analyze the address. ^29^

**Table 1.** Demographic characteristics of respondents.

Respondent characteristic		Frequency	Percentage
Age	Less or equal to 22 years	49	48.5
	More than 22 years	52	51.5
Gender	Male	66	65.3
	Female	35	34.7
Address	Within Kathmandu Valley	19	18.8
	Outside Kathmandu Valley	78	77.2
Year of study	Basic science Year 1	23	22.8
	Basic science Year 2	27	26.7
	Clinical clerkship year 1	24	23.8
	Clinical clerkship year 2	27	26.7
Stay at hostel	Yes	63	62.4
	No	38	37.6
Study background	Health science (Paramedics)	7	6.9
	Intermediate in science (I. Sc.) /A Level	94	93.1

The medical students at PAHS come from two different academic backgrounds: an academic science background where they complete two years of study of science after school level, and from a paramedic background where they must complete three years of study of paramedical health after school level. The academic background of the students is taken in account for this study as shown in Table 1.

### Perceived Stress Scale (PSS)

As shown in Table 2, among 101 students, 55 of them were stressed based on a cut-off score of 28, while 46 of them were not stressed. Consequently, the prevalence of stress in our study was found to be 55%. Notably, female students exhibited a slightly higher prevalence of stress compared to their male counterparts (60% vs 52%) but the difference was not statistically significant (p < 0.05).

**Table 2.** Stress level.

		Not stressed, n (%)	Stressed, n (%)	Total, n	Pearson’s Chi Square test
Gender	Male	32(48)	34(52)	66	p = 0.42
	Female	14(40)	21(60)	35	
Level of study	Basic Science Year 1	8(30)	19(73)	27	p = 0.06
	Basic Science Year 2	9(38)	15(63)	24	
	Clinical Clerkship Year 1	14(52)	13(48)	27	
	Clinical Clerkship Year 2	15(65)	8(35)	23	
Phase of study	Basic Science	17(33)	34(67)	51	p = 0.017
	Clinical Science	29(58)	21(42)	50	
Staying at hostel of medical school	Yes	30(48)	33(52)	63	p = 0.084
	No	16(42)	22(58)	38	
Total		46(46)	55(54)	101(100)	

Similarly, an analysis of stress levels across different academic years reviled a trend with lower stress in senior years. However, the difference among different years was not significant (p > 0.05). In contrast, as shown in Table 2, on regrouping students by the phase of study, a notable finding emerged. Students in initial basic science years were significantly more stressed compared to students in later clinical clerkship years (66.7% vs 42%, with p-value < 0.05).

When considering the accommodation status of students, it was observed that 33 (52.4%) students staying at the PAHS hostel were stressed, while only 22 (57.9%) students not staying in the hostel were stressed. Despite this the difference was not statistically significant (p = 0.10). There was also no significant difference in the presence of stress among local students from within Kathmandu or outside Kathmandu (p > 0.05).

### Medical Student Stressor Questionnaire (MSSQ)

The mean and standard deviation of stressors score are shown in Table 3 below.

**Table 3.** Mean and inference of different domains of stressors.

Stressor	Mean	Standard deviation	Inference
ARS (Academic related stressors)	2.18	0.57	Cause high stress
IRS (Intrapersonal and interpersonal related stressors)	1.88	0.85	Cause moderate stress
TLRS (Teaching and learning-related stressors)	1.85	0.69	Cause moderate stress
SRS (Social related stressors)	1.63	0.66	Cause moderate stress
DRS (Drive and desire related stressors)	1.42	0.8	Cause moderate stress
GARS (Group activities related stressors)	1.72	0.65	Cause moderate stress
Total	**10.68**		

Academic Related Stressors (ARS) scores were significant amongst both male and female students as seen in Table 3. Meanwhile, Social Related Stressors (SRS) was higher among males students, while other stressors were higher among females. When analyzing students’ accommodation status, it was observed that, apart from Intrapersonal and interpersonal Related Stressors (IRS) scores, other stressors were higher in those students staying outside the hostel. However, none of these differences were statistically significant.

The top ten stressors with their mean values are presented in Table 4. Eight of the top ten stressors are ARS. The most common stressors are test examinations, lack of time to review what has been learned, getting poor marks, an extensive amount of content to be learned, and the need to do well (self-expectation).

**Table 4.** Top ten stressors.

	Stressors	Mean values	S.D.	Domain
1	Test examinations	2.74	0.82	ARS
2	Lack of time to review what has been learnt	2.46	0.96	ARS
3	Getting poor marks	2.37	1.11	ARS
4	Large amount of content to be learnt	2.37	1.0	ARS
5	Need to do well (self-expectation)	2.36	1.03	ARS
6	Heavy workload	2.33	0.89	ARS
7	Falling behind in reading schedule	2.19	0.93	ARS
8	Unable to answer the questions from the teachers	2.14	1.07	ARS
9	Lack of guidance from teacher(s)	2.09	1.10	TLRS
10	Verbal or physical abuse by teachers(s)	2.05	1.23	IRS

ARS scores (2.22 vs. 2.14) and GRS scores (1.82 vs. 1.61) were higher in basic science students but other domains were higher in clinical clerkship. Stressors were highest in basic science first-year students while lowest in clinical clerkship second-year students. However, the differences were not statistically significant.

### Brief-COPE

In this study, we observed that the five most common coping strategies used by students during the event of stress were active coping, acceptance, planning, self-distraction, and instrumental support. Conversely, the least common coping mechanism was substance use. The mean scores of coping mechanisms used by students are presented in Table 5.

**Table 5.** Coping strategies adopted by students during stressful events.

Coping mechanism	Overall mean (SD)	Stressed students mean (SD)	Not-stressed students mean (SD)
Active coping	5.86(1.29)	5.69(1.51)	6.06(1.35)
Acceptance	5.67(1.32)	5.61(1.4)	5.73(1.51)
Planning	5.64(1.33)	5.58(1.16)	5.71(1.51)
Self-distraction	5.05(1.37)	5.09(1.41)	5.06(1.33)
Instrumental support	4.85(1.48	4.87(1.45)	4.82(1.53)
Emotional support	4.76(1.26)	4.8(1.43)	4.7(1.02)
Positive reframing	4.75(1.31)	4.7(1.28)	4.8(1.36)
Venting	4.3(1.31)	4.61(1.23)	3.9(1.29)
Religion	4.29(1.56)	4.27(1.71)	4.391.44)
Self-blame	4.01(1.53)	4.41(1.53)	3.52(1.39)
Humor	3.72(1.59)	3.76(1.76)	3.67(1.38)
Behavioral disengagement	3.53(1.4)	3.9(1.45)	3.08(1.36)
Denial	3.42(1.3)	3.49(1.43)	3.329(1.33)
Substance use	2.72(1.3)	2.89(1.46)	2.52(1.27)

There were significant differences in coping strategies used according to gender, phase of the study i.e. basic science or clinical clerkship (p < 0.05), location of accommodation i.e. hostel or outside hostel (p < 0.05), and stress. Male students appear to cope with stress by substance use (p < 0.05). The strategies that were significant among different groups of respondents are presented in Table 6.

**Table 6.** Specific coping strategies among subgroups of respondents.

Coping strategy	Respondent characteristics		Mean value	p-value
Substance use	Gender	Male	3.07	<0.001 *
		Female	2.05	
Active coping	Stay at hostel	Hosteller	6.06	0.043 *
		Day scholar	5.52	
Positive reframing	Phase of study	Basic science	5.07	0.011 *
		Clinical Clerkship	4.42	
Planning	Phase of study	Basic science	5.90	0.048 *
		Clinical Clerkship	4.42	
Religion	Phase of study	Basic science	4.61	0.040 *
		Clinical Clerkship	3.96	
Self-blame	Phase of study	Basic science	4.31	0.044 *
		Clinical Clerkship	3.70	
Active coping	Year of study	Basic science year 1	5.88	0.034 **
		Basic science year 2	6.00	
		Clinical Clerkship year1	6.25	
		Clinical Clerkship year 2	5.21	
Planning	Year of study	Basic science year 1	6.11	0.043 **
		Basic science year 2	5.66	
		Clinical Clerkship year 1	5.66	
		Clinical Clerkship year 2	5.04	

^*^ Student’s Independent Sample T-Test.

^**^ One-Way ANOVA.

## Discussion

We found that 55% of the medical students demonstrated stress above the cut-off value. The prevalence of stress is more among the initial first two years (basic science years) compared to the latter part of the clinical years (66.7% vs. 42.0%). Students reported academic and related stressors as the major sources of stress. In addition, the primary coping strategies used by students during stressful events were active coping, acceptance, planning, self-distraction, and instrumental support. Male students were also using alcohol and substances more frequently than females (p < 0.01) to cope with a stressful situation.

A similar study from Pakistan, which also used PSS to evaluate stress, reported a higher mean PSS score when compared to our study (30.84, SD = 7.01 vs. 27.85 SD = 6.25). ^5^ In a similar study in Manipal, which used GHQ to assess stress, psychological morbidity was 20.9% and it was higher among basic science students. ^9^ A study from Agha Khan University reported that over 90% of the students felt stressed at one time or another during their course. ^17^ Another study from India reported that 73% of the students had perceived stress at one time or another during their medical training. ^18^ Saipanish reported that 61.4% of students in Thai Medical School had experienced some degree of stress as measured by the Thai stress test. ^19^ The amount and severity of stress experienced by medical students appears to vary according to their curriculum, settings of medical schools, and more importantly, the type of psychometric tests used.

In this study female students perceived a higher level of stress as compared to male students however, the difference was not statistically significant. Similarly another study by Cohen *et al*. in the USA showed no significant difference in stress between male and female students. ^13^ However, a similar study in Pakistan reported significantly higher stress among female students compared to their male counterparts. ^5^

More students in basic science years were stressed as compared to clinical years (66.7% vs. 42.0%) which was statistically significant (p = 0.017). It might be because they have adapted to the system and setting of PAHS. A study done at Manipal, another Nepali medical school, showed the highest level of stress in the first-year basic science students. At PAHS, stress levels decreased with the increase in the year of study. At Manipal, however, there was less stress in the second year of basic science but higher stress in the clinical years of study. ^9^ Similar results were reported in studies from Pakistan, Thailand, and India. ^13^^,^^17^^,^^18^ We do not have tools to evaluate this discrepancy, but a plausible explanation might be different academic settings between these institutions.

A study that evaluated the correlation between stress and academic performance in the first two years of medical school showed a negative correlation between stress and academic performance. ^8^ Though we did not evaluate academic performance in our study, we expect it might be similar here as well.

The mean score of MSSQ at our institution was 1.88 (moderate stress) whereas it was 3.9 (severe stress) at Taibah University, Saudi Arabia. ^20^ The major source of high stress among the students in our studies was ARS, which was similar to a study done in Xavier University School of Medicine, Netherlands, that showed high ARS and GRS. ^21^ In Saudi Arabia, ARS and IRS were high. ^20^ Though the overall TLRS and IRS score caused moderate stress, lack of guidance from teachers and verbal abuse from teachers caused high stress in PAHS. The major stressors at the Pakistani Medical School were also of the academic and psychosocial domains. ^5^

Our study showed that academic stress is higher among basic science students. However, other studies done in Pakistan, Saudi Arabia, Netherlands, and Manipal have shown that academic stress is higher in the students in their clinical years. ^3^^,^^4^^,^^9^^,^^20^

This study shows test examinations, lack of time to review what has been learned, and, a large amount of content as main academic stressors. This was similar to a study done in Manipal, where the most common causes of stress were dissatisfaction with lecture classes, the vastness of the academic curriculum, and the frequency of examinations. ^9^ A study conducted in the United Arab Emirates (UAE) also showed that the major stressors among the students were the frequency of examinations, time management, and academic workload. ^22^

Coping strategies are behavioral and psychological efforts that apply to master, tolerate, reduce, or minimize stressful events. ^23^ The primary coping strategies used by students during stressful events were active coping, acceptance, planning, self-distraction, and instrumental support. Students from Manipal also used similar coping strategies along with positive reframing and emotional support. ^9^ Similar studies from UAE, India, Netherlands, and UK also showed similar results. ^21^^–^^25^ Although the coping strategies were different among males and females, the only significant difference in strategy was substance use, which was higher in males (p < 0.01): a finding similar to a study in Glasgow University, UK. ^25^ In India, another statistically significant difference was observed, females used meditation as a coping strategy. ^24^

We saw that stressed students used venting and self-blame, similar to the study done in Manipal. ^9^ The medical students from Brazil who were stressed used escape-avoidance tactics more than non-stressed students. ^26^ Pakistani post graduate students who were using maladaptive coping styles were more likely to be stressed. ^27^ However, it is not clear whether there is a cause-effect relation between avoidant coping strategies and stress.

Substance use was the least used coping mechanism by students, which may have been under-reported despite confidentiality and anonymity of the response was assured. There were low incidences of alcohol/substance use in Manipal as well as in Pakistan, which may be due to the social stigmata associated with alcohol use in these societies. ^9^^,^^27^ But studies from the UK suggest the use of alcohol, tobacco, and drugs as common strategies. ^28^^,^^29^ The cultural variation between the two societies may have contributed to the difference.

All stressor domains of MSSQ and total MSSQ positively correlated with the PSS score, which was significant, except for DRS, as shown in Table 3. It suggests that students with more stressors in (most of the domains) MSSQ are also likely to perceive more stress. The correlation was moderate between total PSS score and ARS, GRS, and total MSSQ while it was weak within total PSS, IRS, SRS, DRS, and TLRS.

Our study also showed that students with high stress were likely to have maladaptive coping strategies. The correlation was moderate. Though the difference was not statistically significant, the total PSS scores negatively correlated with adaptive coping strategies. This indicates that students might have decreased stress due to adaptive coping.

### Limitations

Our findings may not be generalizable to other medical schools in Nepal since PAHS has a unique teaching-learning strategy. The cross-sectional design of our study is another limitation since associations could not be calculated. Prospective studies are necessary to study the associations between stressors and the incidence of stress. Since we did not evaluate the level of stress with academic performance, we cannot comment on the association between the two. The questionnaires were used as it is in the English language, potentially causing issues with students as this may not be their first language. The systematic translation, cultural validation, and adaptation in the local language of Nepalese medical students was not done.

## Conclusion

Stress level among PAHS students was high, with academic stress being the predominant factor. Further studies can be done to evaluate the effects of stress on the academic outcomes of students. Notably the stress level was highest among the first-year students. This has a strong implication to provide a support mechanism for the first-year students. Strategies to enhance teacher-student communication could be implemented in addressing the issue. Additionally, since stressed students were more likely to use maladaptive coping strategies like behavioral disengagement, substance use, venting, denial, and self-blame, measures should be taken to support students to adopt a more positive and healthy coping approach.

## Data availability

### Underlying data

Figshare: Underlying data of “Perceived Stress, Sources of Stress and Coping Strategies among Undergraduate Medical Students of Nepal”. https://doi.org/10.6084/m9.figshare.c.5757833.v1. ^29^

The project contains the following underlying data:

- Raw data of Stress.xlsx

### Extended data

Figshare: Underlying data of “Perceived Stress, Sources of Stress and Coping Strategies among Undergraduate Medical Students of Nepal”. https://doi.org/10.6084/m9.figshare.c.5757833.v1. ^29^

The project contains the following extended data:

- Questionnaire of Stress.docx

Data are available under the terms of the Creative Commons Attribution 4.0 International license (CC-BY 4.0).

## Ethics approval and consent to participate

This study was approved by the Institutional Review Committee (Ethical Committee) of Patan Academy of Health Sciences (PAHS), Kathmandu, Nepal with ref no: std1506111071. Written informed consent was obtained from all participants.

## Consent for publication

Written informed consent was obtained from the participants.

## Authors’ contributions

UP, AP, RS, and SK first planned conception and designed this research. UP, AP, RS, and SK did the data collection. All six authors, UP, AP, RS, SK, SAY, and KM worked for data analysis and interpretation. UP, AP, RS, and SK wrote the first draft of the manuscript. SAY and KM did further revision and editing of the manuscript. All six authors, SAY, SP, UP, AP, RS, SK, and KM critically revised the article. And final approval of the version to be published was also done by all six authors.
