# Supplementary material for: Perceived stress, sources of stress and coping strategies among undergraduate medical students of Nepal: a cross-sectional study
Source: F1000Res. 2024 Jan 3;11:167. Originally published 2022 Feb 10. [Version 3] doi: 10.12688/f1000research.75879.3 (PMC11384203; doi:10.12688/f1000research.75879.3)
Supplement: Peer review history

# Reviewer report: Reviewer response for version 3

**DOI:** 10.5256/f1000research.156923.r277561

**Published:** 2024-09-09

**Authors:** Prakash Banjade

**Affiliations:** 1 Manipal College of Medical Sciences, Pokhara, Nepal

The authors have revised the manuscript, addressing statistical explanations and clarifying results. These improvements have strengthened the study's validity and clarity of presentation. Overall, this manuscript offers significant contributions to understanding stress and coping mechanisms among medical students in Nepal. Its findings are relevant to the audience interested in medical education and student mental health.

Is the work clearly and accurately presented and does it cite the current literature?

Yes

If applicable, is the statistical analysis and its interpretation appropriate?

I cannot comment. A qualified statistician is required.

Are all the source data underlying the results available to ensure full reproducibility?

Yes

Is the study design appropriate and is the work technically sound?

Yes

Are the conclusions drawn adequately supported by the results?

Yes

Are sufficient details of methods and analysis provided to allow replication by others?

Yes

Reviewer Expertise:

Pulmonology, Cardiovascular diseases

I confirm that I have read this submission and believe that I have an appropriate level of expertise to confirm that it is of an acceptable scientific standard.

# Reviewer report: Reviewer response for version 2

**DOI:** 10.5256/f1000research.155814.r206199

**Published:** 2023-09-29

**Authors:** Alicia Fournier

**Affiliations:** 1 Universite de Bourgogne, Dijon, Bourgogne-Franche-Comté, France

I would like to thank the authors for the revisions made to their manuscripts. Unfortunately, these revisions are not sufficient. Further revisions are required for indexing.

 1. In the abstract

 1.1 The authors should mention the number of participants included in the analyses.

 1.2 In the results section, the authors mention a difference in perceived stress between first-year students and trainees. It is not clear whether the results are for the whole sample or just the first-year students. To be reworded.

 1.3. In the conclusion, the authors mention that the most stressed students use maladaptive coping, but there is nothing to this effect in the results section.

 1.4. I would remove the response rate. 

 The introduction has been improved. My thanks to the authors.

 2. In the material section, how did the authors categorise the MSSQ scores? Do the classes come from the literature? If so, mention the reference.

 3. The statistical analysis section needs to be greatly improved. The authors only mention having carried out descriptive tests and Pearson correlations, which is not the case. In fact, the authors performed chi2 tests, ANOVAs and t-tests in addition to correlation analyses. I would also like to point out that the results of these correlation analyses never appear in the results section, despite a discussion of these correlations in the discussion section. 

 4. In the results section,

 4.1. Do not refer to prevalence for the PSS scale. It is a question of frequency, given the size of your sample. To talk about prevalence, the authors would have had to survey virtually all the university's students.

 4.2 The authors should reword the sentence "Similarly, an analysis of stress levels across different academic years reviled a trend with lower stress in senior years." Indeed, it is confusing; it could suggest that statistical analyses have been carried out revealing significant differences between each time.

 4.3 In the paper as a whole, the authors cannot report differences between individuals if, statistically, there are none. For example "Academic Related Stressors (ARS) scores were significant amongst both male and female students as seen in Table 3. Meanwhile, Social Related Stressors (SRS) was higher among male students, while other stressors were higher among females. When analyzing students' accommodation status, it was observed that, apart from Intrapersonal and interpersonal Related Stressors (IRS) scores, other stressors were higher in those students staying outside the hostel. However, none of these differences were statistically significant." Everything mentioned is wrong because the authors mention an absence of significance for these differences between the groups. This occurs numerous times in the paper and should be corrected. Furthermore, Table 3 shows absolutely no comparison between the groups. This same table is referred to in the discussion as a correlation table. The authors should correct this problem.

 4.4. Remove the "s" from male in the above paragraph.

 4.5. The authors did not compare stressed and unstressed students on the MSSQ stress factors, which is a pity because it would be very informative about the associations between stress factors and perceived stress.

 4.6. The section on Brief-COPE needs to be revised. The paragraph "There were significant differences in coping strategies used according to gender, phase of the study i.e. basic science or clinical clerkship (p < 0.05), location of accommodation i.e. hostel or outside hostel (p < 0.05), and stress. Male students appear to cope with stress by substance use (p < 0.05). The strategies that were significant among different groups of respondents are presented in Table 6." is not clear. What were the authors trying to achieve? What do the different groups correspond to? Table 6 is not at all helpful. The authors have carried out a whole host of analyses and only report the significant ones? Honestly, I don't understand this table at all.

 4.7. It should be mentioned that compared to women “Male students appear to cope with stress by substance use (p < 0.05)”.

 4.8. In the abstract, the authors say that stressed students use more maladaptive strategies, but I don't see this in the results section. Moreover, according to Table 5, stressed students mainly use the active coping, acceptance, planning, self-distraction, and instrumental support. Are these maladaptive strategies? Did the authors compare the strategy scores of the stressed and unstressed groups?

 Moreover, the authors don't mention maladaptive and adaptive strategies, which is normal because a strategy can be adapted in certain circumstances and maladaptive in others. The authors should review this concept.

 5. In the discussion section,

 5.1. Authors should not mention statistical results.

 5.2. As mentioned earlier, if the authors do not find statistical differences, they cannot mention differences, even if the numbers might suggest it. What matters are the statistical results!

 5.3. I cannot find this result in the "results" section. « We saw that stressed students used venting and self-blame”.

 5.4. “All stressor domains of MSSQ and total MSSQ positively correlated with the PSS score, which was significant, except for DRS, as shown in Table 3. It suggests that students with more stressors in (most of the domains) MSSQ are also likely to perceive more stress. The correlation was moderate between total PSS score and ARS, GRS, and total MSSQ while it was weak within total PSS, IRS, SRS, DRS, and TLRS.” Idem, I cannot find this result in the "results" section. Once again, Table 3 does not correspond to what the authors indicate.

Is the work clearly and accurately presented and does it cite the current literature?

Partly

If applicable, is the statistical analysis and its interpretation appropriate?

Partly

Are all the source data underlying the results available to ensure full reproducibility?

Yes

Is the study design appropriate and is the work technically sound?

Partly

Are the conclusions drawn adequately supported by the results?

Yes

Are sufficient details of methods and analysis provided to allow replication by others?

No

Reviewer Expertise:

Stress, Emotion regulation, Quality of life at work, Interoception

I confirm that I have read this submission and believe that I have an appropriate level of expertise to confirm that it is of an acceptable scientific standard, however I have significant reservations, as outlined above.

# Response

**Published:** 2023-10-02

**Authors:** Saroj Adhikari Yadav

**Affiliations:** Patan Academy of Health Sciences School of Medicine, Nepal

Dear Reviewer,

 We would like to express our gratitude for your thoughtful and comprehensive review of our manuscript. Your feedback has been invaluable in improving the quality and clarity of our work. We have addressed each of your concerns and also incorporated other reviewers' and study mentors' feedback in the revised version of the manuscript.

 In the abstract: The number of participants included in the analyses has been mentioned. The reference group in the results section has been clarified. Statements in the conclusion now align with the corresponding results.

 In the Method section: Information on the categorization of the MSSQ scores has been done in a standard manner, and relevant references have been included. The manuscript now accurately reflects the statistical tests we performed, and the results of these analyses have been appropriately presented in the results section.

 In the results section: The Brief-COPE section has been revised to provide a clearer explanation of our goals, and the Table 6 explanation has been rewritten for better clarity. The wording has been adjusted and rephrased for clarity. References to statistical significance have been corrected. We have compared stressed and unstressed students on the MSSQ stress factors and reported the findings as relevant. We have clarified our use of the terms "maladaptive" and "adaptive" strategies and reviewed the concept as suggested.

 In the discussion section: Statistical results have been kept only in necessary sentences of the discussion for clarity of the readers. Differences are only mentioned when statistical significance is found or is required to be mentioned due to statistically significant differences in other studies but not ours (like stressed based on gender). The mentioned result and its presence in the results section have been reviewed.

 We can see your review has significantly improved the manuscript's overall quality and accuracy. Your detailed and meticulous feedback has been instrumental in this process, and we genuinely appreciate your time and effort in reviewing our work. Thank you for your help and support.

 Sincerely,

 Dr Adhikari Yadav, MD.

# Reviewer report: Reviewer response for version 2

**DOI:** 10.5256/f1000research.155814.r206200

**Published:** 2023-09-26

**Authors:** Bijit Biswas

**Affiliations:** 1 Department of Community and Family Medicine, All India Institute of Medical Sciences, Deoghar, Jharkhand, India

The revisions made by the authors were satisfactory..

Is the work clearly and accurately presented and does it cite the current literature?

Yes

If applicable, is the statistical analysis and its interpretation appropriate?

Partly

Are all the source data underlying the results available to ensure full reproducibility?

Yes

Is the study design appropriate and is the work technically sound?

Partly

Are the conclusions drawn adequately supported by the results?

Yes

Are sufficient details of methods and analysis provided to allow replication by others?

Partly

Reviewer Expertise:

NA

I confirm that I have read this submission and believe that I have an appropriate level of expertise to confirm that it is of an acceptable scientific standard.

# Reviewer report: Reviewer response for version 1

**DOI:** 10.5256/f1000research.79808.r168111

**Published:** 2023-05-15

**Authors:** Alicia Fournier

**Affiliations:** 1 Universite de Bourgogne, Dijon, Bourgogne-Franche-Comté, France

The objective of this paper was to identify the perceived intensity of stress and its sources among medical students in Nepal and to identify coping strategies used to deal with this stress. This exploratory cross-sectional study was conducted on 101 Nepalese students and provides new information on the origin of perceived stress.

 Although it is important to take care of the health of medical students, this study remains too descriptive. The authors could have used moderation analyses to study the effects of coping strategies on the stressor/perceived stress factor relationship.

 Regarding the introduction, the authors do not sufficiently develop the innovative aspect of their study. For example, the authors highlight an innovative approach to teaching and learning in the school where the study was conducted. The authors should develop this approach and compare their results with schools in Nepal where there is no such teaching.

 Also, the authors should further develop what the first cycle is. Throughout the paper, I did not understand the difference between students in basic sciences and those in clinical placement. It was only thanks to the first line of the discussion that I understood the difference. Additionally, mixing early and late cycle students in the analyses is not relevant. Stress factors are not the same, so wouldn't it be beneficial for the authors to compare these two groups of students throughout the paper? Furthermore, Table 1 confuses me: what is the context of the study? Are they all medical students or are there also paramedical students?

 Moreover, the statistical analyses are not sufficiently developed and well reported. For example, the analyses carried out to compare the participants are only mentioned in a table note. The authors should mention this in the statistical section. Additionally, they do not explain why they performed t-tests in some cases and ANOVAs in others. The result section needs to be rewritten. This part remains too vague and difficult to understand. There is a lack of a guiding thread so that the reader doesn't get lost. Descriptive tables of all variables are also missing.

 I also draw the authors' attention to the choice of the 28 cutoff for the PSS-14. I would like to remind them that this tool is not a diagnostic tool and that it would be preferable to use it as a continuous score. The cutoff of 28 is an arbitrary score used in papers but remains unvalidated. The authors should be cautious in their interpretation.

Is the work clearly and accurately presented and does it cite the current literature?

Partly

If applicable, is the statistical analysis and its interpretation appropriate?

Partly

Are all the source data underlying the results available to ensure full reproducibility?

Yes

Is the study design appropriate and is the work technically sound?

Partly

Are the conclusions drawn adequately supported by the results?

Yes

Are sufficient details of methods and analysis provided to allow replication by others?

No

Reviewer Expertise:

Stress, Emotion regulation, Quality of life at work, Interoception

I confirm that I have read this submission and believe that I have an appropriate level of expertise to confirm that it is of an acceptable scientific standard, however I have significant reservations, as outlined above.

# Response

**Published:** 2023-09-08

**Authors:** Saroj Adhikari Yadav

**Affiliations:** Patan Academy of Health Sciences School of Medicine, Nepal

Dear Reviewer,

 Thank you for your comprehensive review and invaluable feedback on our manuscript. We greatly appreciate your time and insights, which have been instrumental in refining our work.

 We are pleased to inform you that we have diligently addressed your suggestions and made the required edits to enhance the manuscript's quality and clarity. Here's a brief overview of how we incorporated your valuable input:

1. **Statistical Analysis**: We have provided a more detailed explanation of the statistical analyses used in the methods section, including the rationale for choosing specific tests. Additionally, we've included a table summarizing the descriptive statistics of all variables.
2. **Introduction**: We have expanded on the innovative aspects of our study, particularly the unique teaching and learning approach at the school where the research was conducted. We've also clarified the distinctions between students in basic sciences and those in clinical placement.
3. **Comparison of Student Groups**: We now focus our analyses on early and late-cycle students, recognizing the differences in stress factors. This change will provide more meaningful insights. Your further recommendations can be meaningful for us when we plan our next research project.
4. **PSS-14 Cutoff**: We acknowledge your concern regarding the PSS-14 cutoff and have revised our interpretation accordingly, emphasizing its use as a continuous score, though 50% cutoff is used in other similar articles as well and is a norm in biostat.

These revisions, guided by your feedback, and our mentors' suggestions, have significantly improved the manuscript. We remain committed to ensuring that our research meets the highest standards, and your feedback has been invaluable in achieving this goal. We look forward to your continued evaluation and input as we work towards producing a more robust and insightful contribution to the field.

 Once again, thank you for your dedication to improving our research. Your efforts are sincerely appreciated.

 Warm regards,

 -Dr Adhikari Yadav

# Reviewer report: Reviewer response for version 1

**DOI:** 10.5256/f1000research.79808.r147808

**Published:** 2022-08-26

**Authors:** Bijit Biswas

**Affiliations:** 1 Department of Community and Family Medicine, All India Institute of Medical Sciences, Deoghar, Jharkhand, India

The study was aimed at assessing severity, sources of stress, and coping strategies among medical students of a non-Western low-income country in South Asia. The article has several major issues, especially in the methodology.

 Abstract

1. In the abstract please avoid terms like "non-Western low-income country from South Asia", be specific (i.e, Nepal)
2. Please add statistical findings in the abstract (i.e, correlation coefficient with p values) along with text to enrich it.

Introduction:

1.  Avoid 2-3 sentence paragraphs
2. No previous study is not a sufficient rationale. Kindly state other causes.

Methods:

1. Why was 50% prevalence assumed for sample size calculation? If no prior study on this topic in Nepal author may cite other studies conducted in the sub-continent or region for sample size calculation.
2. Kindly elaborate on 2 stages of stratified random sampling used in the study. preferably add a flowchart.
3. If 106 was the minimum sample size then how could statistical tests be done in a lower sample (i.e, 101)?
4. Report validity and reliability measures used for the tools used in the study (i.e, Cronbach's alpha).
5. Was the questionnaire administered in English or the local language? If local language authors had to prevalidate translation before using them for the study.
6. Statistical analysis portion did not state the exact tests used for the study in some cases (i.e, ANOVA)

Results, Discussion, and Conclusion seem ok.

 References did not follow any particular style (i.e, Vancouver)

 The article cited very old references (>10 years) which might not be relevant now.

Is the work clearly and accurately presented and does it cite the current literature?

Yes

If applicable, is the statistical analysis and its interpretation appropriate?

Partly

Are all the source data underlying the results available to ensure full reproducibility?

Yes

Is the study design appropriate and is the work technically sound?

Partly

Are the conclusions drawn adequately supported by the results?

Yes

Are sufficient details of methods and analysis provided to allow replication by others?

Partly

Reviewer Expertise:

Chronic Disease Epidemiology, Mental Health, Occupational Health

I confirm that I have read this submission and believe that I have an appropriate level of expertise to state that I do not consider it to be of an acceptable scientific standard, for reasons outlined above.

# Response

**Published:** 2023-09-08

**Authors:** Saroj Adhikari Yadav

**Affiliations:** Patan Academy of Health Sciences School of Medicine, Nepal

Dear Reviewer,

 We extend our sincere gratitude for your meticulous review of our manuscript. Your insightful feedback, alongside the contributions from fellow reviewers and our esteemed mentors, has been instrumental in enhancing the quality of our work.

 Addressing your suggestions, we have diligently incorporated the following revisions into the abstract:

- In response to your recommendation, we have included the specific name of the country in the abstract, thereby providing a more comprehensive contextualization of our study.
- We have pruned the abstract to exclude insignificant findings, ensuring that it now succinctly encapsulates the essence of our research.
- The logical merging of smaller paragraphs has been executed, enhancing the abstract's coherence and flow.
- We have redefined the rationale, offering readers a clearer understanding of the motivations behind our study.
- Furthermore, we have elaborated on the two stages of stratified random sampling, with the aim of facilitating easier comprehension for our readers.
- The administration of the questionnaire in English has been more comprehensively explained, providing essential context to our methodology.
- We have ensured that the appropriate statistical analysis methods employed are adequately mentioned.

Regarding the prevalence assumption and sample size, we appreciate your consideration of the statistical norms of using 50% as a baseline, and the 10% non-response rate was added while calculating a sample size of 106. Following a thorough review of the biostatistical recommendations, we have decided to retain these aspects as they are.

 Once again, we would like to express our gratitude for your invaluable input, which has undoubtedly enriched our research. Your dedication to the peer review process is greatly appreciated.

 Best regards,

 Dr. Adhikari Yadav
